# Expression of Nerve Growth Factor (NGF), TrkA, and p75^NTR^ in Developing Human Fetal Teeth

**DOI:** 10.3389/fphys.2016.00338

**Published:** 2016-08-03

**Authors:** Thimios A. Mitsiadis, Pierfrancesco Pagella

**Affiliations:** Orofacial Development and Regeneration, Institute of Oral Biology, Center for Dentistry (ZZM), University of ZurichZurich, Switzerland

**Keywords:** human, tooth, development, nerve growth factor, odontoblast, ameloblast, TrkA, p75^NTR^

## Abstract

Nerve growth factor (NGF) is important for the development and the differentiation of neuronal and non-neuronal cells. NGF binds to specific low- and high-affinity cell surface receptors, respectively, p75^NTR^ and TrkA. In the present study, we examined by immunohistochemistry the expression patterns of the NGF, p75^NTR^, and TrkA proteins during human fetal tooth development, in order to better understand the mode of NGF signaling action in dental tissues. The results obtained show that these molecules are expressed in a wide range of dental cells of both epithelial and mesenchymal origin during early stages of odontogenesis, as well as in nerve fibers that surround the developing tooth germs. At more advanced developmental stages, NGF and TrkA are localized in differentiated cells with secretory capacities such as preameloblasts/ameloblasts secreting enamel matrix and odontoblasts secreting dentine matrix. In contrast, p75^NTR^ expression is absent from these secretory cells and restricted in proliferating cells of the dental epithelium. The temporospatial distribution of NGF and p75^NTR^ in fetal human teeth is similar, but not identical, with that observed previously in the developing rodent teeth, thus indicating that the genetic information is well-conserved during evolution. The expression patterns of NGF, p75^NTR^, and TrkA during odontogenesis suggest regulatory roles for NGF signaling in proliferation and differentiation of epithelial and mesenchymal cells, as well as in attraction and sprouting of nerve fibers within dental tissues.

## Introduction

The roles of nerve growth factor (NGF) in the development, survival and maintenance of selected group of neurons of the peripheral and central nervous system have been thoroughly evaluated in the last few decades (Chao, 2003; Lu et al., 2005; Reichardt, 2006; Ichim et al., 2012; Lewis and Carter, 2014). Dependence of neurons on NGF varies as a function of the stage of development. Additional members of the NGF-related family of neurotrophic molecules include brain-derived neurotrophic factor (BDNF), neurotrophin-3 (NT-3), neurotrophin-4 (NT-4; also known as NT-4/5 or NT-5), and neurotrophin-6 (NT-6; Chao, 2003; Lu et al., 2005; Reichardt, 2006; Lewis and Carter, 2014). NGF-related neurotrophins (NTFs) support the survival and outgrowth of various neuronal populations (Reichardt, 2006; Ichim et al., 2012). Target cells for NTFs bear specific cell-surface receptors and their presence is indicative of a potentially responsive cell (Chao, 2003; Reichardt, 2006). Two binding affinities of NTFs to their receptors, one high, the other low, have been described. A transmembrane glycoprotein called low-affinity NGF receptor (p75^NTR^) binds all NTFs with low-affinity (Radeke et al., 1987; Chao, 1992; Reichardt, 2006). However, it is still unclear whether the low-affinity form is capable of mediating all biological responses of NTFs. The products of the tyrosine kinase *trk* family of proto-oncogenes bind also NTFs, and are components of the high-affinity receptor. The *trk* gene family is formed of three characterized genes, *trkA, trkB, trkC* (Chao, 1992; Barbacid, 1994; Reichardt, 2006; Lewis and Carter, 2014). The *trkA* gene encodes a 140 kDa glycoprotein with a tyrosine kinase activity, which functions as a NGF receptor (Klein et al., 1991). Functional high-affinity NGF binding requires either co-expression and binding to both p75^NTR^ and TrkA (Kaplan et al., 1991) or binding to dimers of TrkA (Chao, 2003; Reichardt, 2006).

Novel roles for NTFs in embryonic development are proposed by the presence of p75^NTR^ and Trk receptors during organ morphogenesis and differentiation of non-neuronal cells (Chesa et al., 1988; Yan and Johnson, 1988; Represa and Bernd, 1989; von Bartheld et al., 1991; Nakamura et al., 2007; Di Girolamo et al., 2008; Truzzi et al., 2011; Tomellini et al., 2015). Indeed, expression of both p75^NTR^ and NGF in the developing rodent teeth (Yan and Johnson, 1988; Byers et al., 1990; Mitsiadis et al., 1992, 1993; Mitsiadis and Luukko, 1995) suggests that NTFs play multiple roles in odontogenesis, dental cell function, and tooth homeostasis. The tooth develops as a result of sequential and reciprocal interactions between the oral ectoderm and the cephalic neural crest-derived mesenchyme (Mitsiadis and Graf, 2009). Differentiation of tooth-specific cells gives rise to the mesenchymal-derived odontoblasts that produce the organic matrix of dentine, and the epithelial-derived ameloblasts that elaborate the enamel matrix proteins. In rodents, concomitant expression of p75^NTR^ and NGF in dental mesenchyme is correlated with odontoblast differentiation, whereas in dental epithelium their co-expression corresponds mostly to proliferative phenomena (Mitsiadis et al., 1992, 1993; Mitsiadis and Luukko, 1995). These findings indicate that NGF may be implicated in morphogenetic and mineralization events by affecting either proliferation or differentiation of dental cells (Mitsiadis et al., 1993).

Although numerous studies are undertaken in rodents to understand the role of NGF signaling in tooth development and regeneration, only limited studies exist in humans. Previous data have focused only on the localization of p75^NTR^ in both embryonic and adult teeth. These reports have shown that in the developing fetal teeth p75^NTR^ is expressed transiently in both dental papilla mesenchyme and inner dental epithelium (Christensen et al., 1993), whereas in adult teeth the receptor is present only in unmyelinated axons and Schwann cells of the pulp (Maeda et al., 1992). To date, there is no available data on the distribution of both NGF and TrkA proteins in the developing human teeth. The present study was conducted to localize areas and specific dental cells that express NGF, p75^NTR^, and TrkA in developing human teeth, in order to better understand the mode of NGF action in dental tissues.

## Materials and methods

### Embryonic tissues

#### Tissues

Human fetal tissues were obtained from legal abortions. The material comprised teeth from 19 fetuses (5–23 gestational weeks). The gestation age was estimated from the fetal foot length and from the last menstruation of the mother. Embryos were non-infected, and all tissues were both macroscopically and microscopically normal. The fetuses were immediately fixed in 10% buffered formalin for 48 h to 5 days according to the fetus size. Maxillary and mandibular jaws from 5 to 15 weeks old embryos and fetuses were embedded in Paraplast at 56°C, while the samples ranged in age from 19 to 23 gestational weeks (g.w.) were decalcified for 3 weeks in formic acid/10% formalin prior to embedding in Paraplast. Four to six micrometer thick sections were used for immunohistochemistry. This study was carried out in compliance with the French legislation, after approval of the Regional Ethics Committee of Development and Reproduction of the U.F.R. of Medicine of Reims-France (INSERM 314 Reims).

### Materials

#### Antibodies

Preparation, purification and characterization of polyclonal anti-NGF antibodies have been described (Mitsiadis et al., 1992, 1993). Affinity purified mouse anti-human p75^NTR^ monoclonal antibody was the kind gift of Dr. E. M. Johnson Jr. and Dr. C. Osborn (St. Louis, USA). The purification and characterization of the 20.4 antibody, which recognizes the human p75^NTR^, has been already described (Ross et al., 1984; Grob et al., 1985; Chesa et al., 1988). Polyclonal TrkA antibody was purchased from Abcam (ab76291).

#### Chemicals

Vector Vectastain ABC kit was purchased from Biosys (Compiègne, France). Other chemicals were obtained from Sigma (St. Louis, MO, USA).

### Immunohistochemistry

Immunoperoxidase staining was performed as previously described (Mitsiadis et al., 1992, 1993, 2003). Briefly, the sections were deparaffinized, treated with 0.4% pepsin, exposed to a 0.3% solution of hydrogen peroxide in methanol, and then incubated overnight at 4°C in a humid atmosphere either with polyclonal anti-NGF and anti-TrkA antibodies diluted 1:300 in PBS containing 0.2% BSA, or with the monoclonal anti-p75^NTR^ antibody 20.4 (1:1000 dilution). Positive peroxidase staining produces red/brown color on light microscopy. After staining the sections were mounted with Eukit. In control sections the antibodies were omitted.

## Results

**Figure 1:**
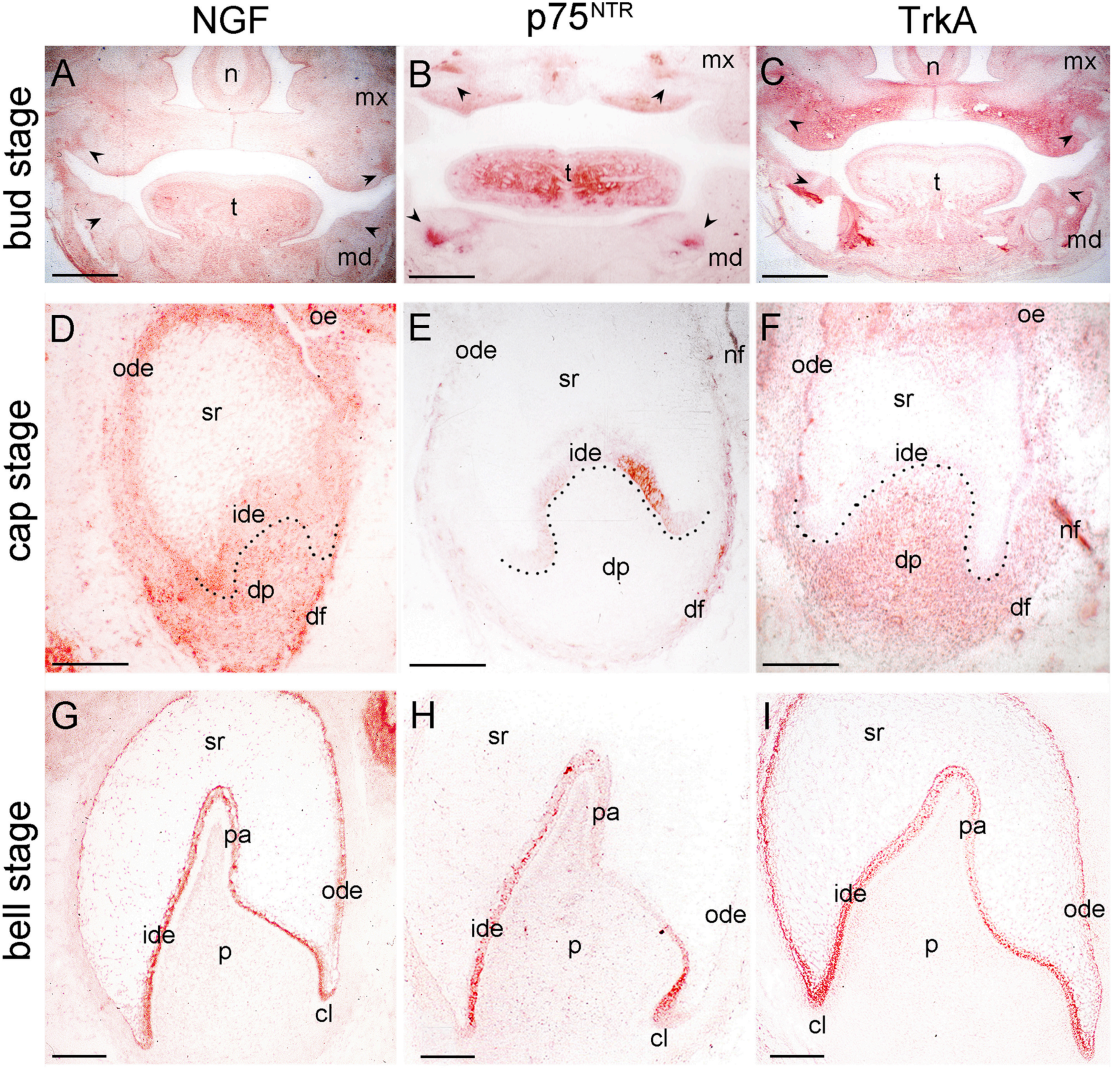
**Distribution of NGF, p75^**NTR**^, and TrkA proteins in developing human teeth. (A–C)** Frontal sections of human fetal head with tooth germs at bud/early cap stage (arrowheads). Immunostaining is seen in red color. **(D–F)** Tooth germs at the late cap stage. NGF **(D)**, p75^NTR^ **(E)**, and TrkA **(F)** immunoreactivities. Note the staining in the nerve fibers (nf) in the dental follicle (df). **(G–I)** Tooth germs at the early bell stage. NGF **(G)**, p75^NTR^ **(H)**, and TrkA **(I)** immunostainings. Dotted lines represent the border between the dental epithelial and mesenchymal components. Abbreviations: cl, cervical loop; dp, dental papilla; ide, inner dental epithelium; md, mandible; mx, maxilla; n, nose; nf, nerve fibers; ode, outer dental epithelium; oe, oral epithelium; p, dental pulp; pa, preameloblasts; sr, stellate reticulum; t, tongue. Scale bars: **(A–C)** 1 mm, **(D–I)** 100 μm.

**Figure 2:**
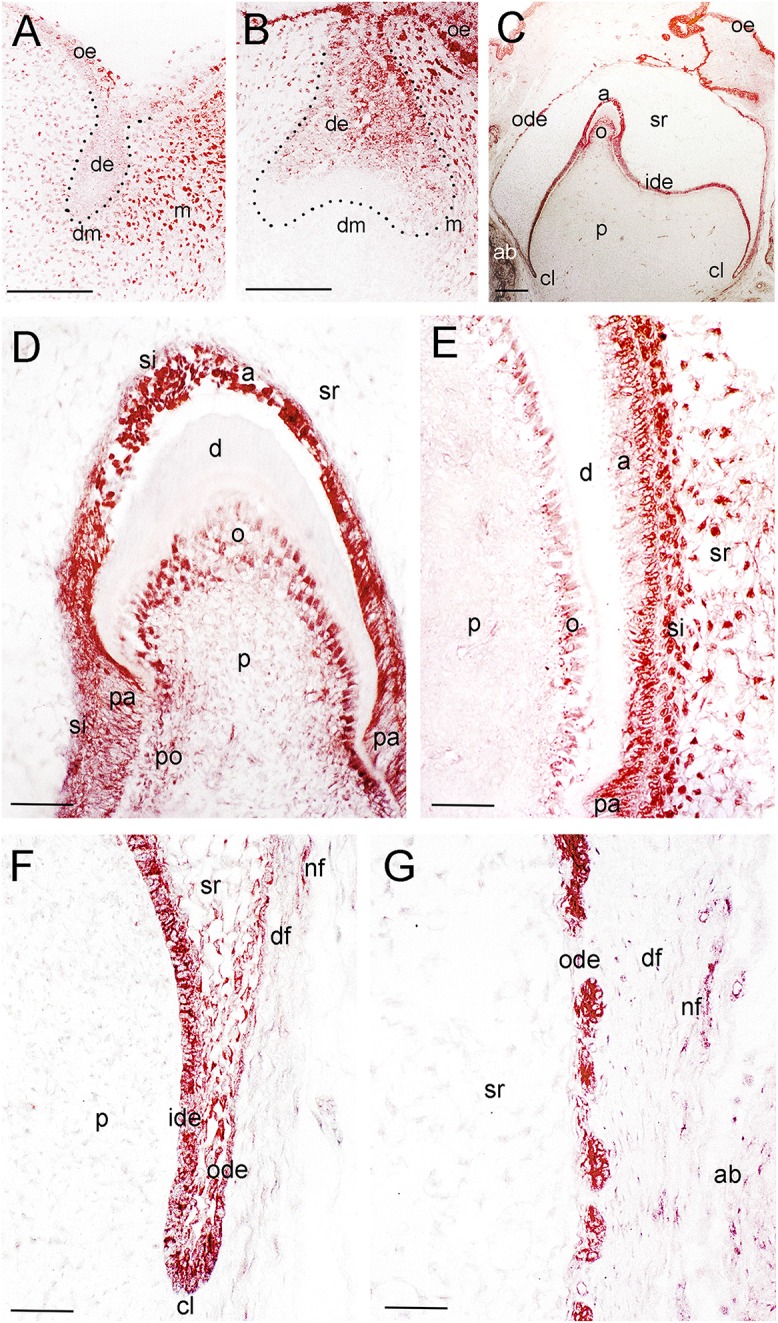
**Distribution of the NGF protein in developing human teeth. (A)** NGF immunoreactivity (red color) in a tooth germ at the bud stage. **(B)** NGF expression in a tooth germ at the early cap stage. **(C–G)** NGF staining in a tooth germ at the late bell stage. **(D–G)** Higher magnifications of **(C)**, representing the tip of the cusp area **(D)**, an area of the flanks of the forming tooth crown **(E)**, the cervical loop territory **(F)**, and the dental follicle territory **(G)**. Dotted lines represent the border between the dental epithelial and mesenchymal components. Abbreviations: a, ameloblasts; ab, alveolar bone; cl, cervical loop; d, dentinee; de, dental epithelium; df, dental follicle; dm, dental mesenchyme; ide, inner dental epithelium; m, mesenchyme; nf, nerve fibers; o, odontoblasts; ode, outer dental epithelium; oe, oral epithelium; p, dental pulp; pa, preameloblasts; po, preodontoblasts; si, stratum intermedium; sr, stellate reticulum. Scale bars: **(A–C)** 100 μm, **(D–G)** 25 μm.

**Figure 3:**
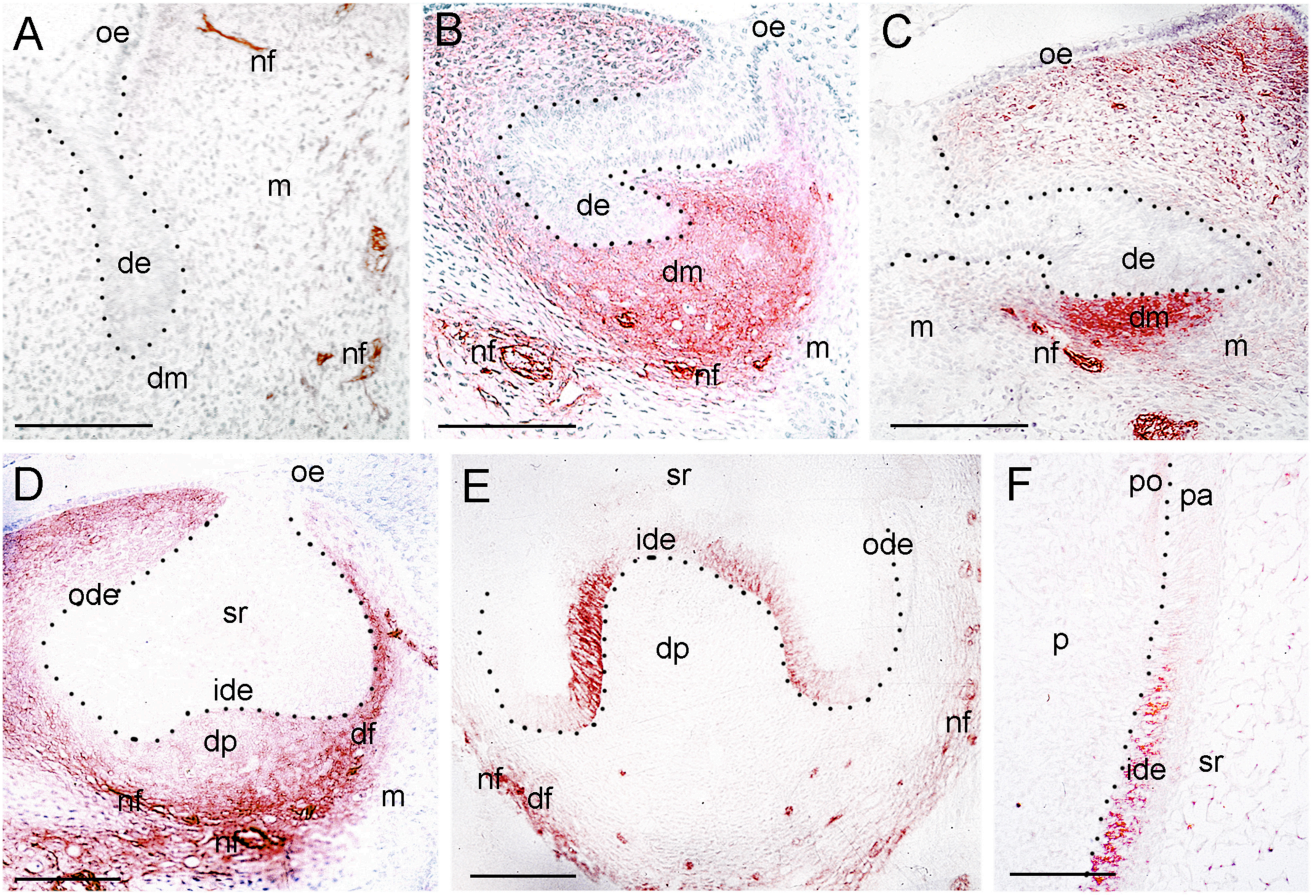
**Expression of p75^**NTR**^ during tooth development. (A)** A tooth germ at the bud stage. Note the p75^NTR^ immunoreactivity (red color) in the nerve fibers (nf) approaching the tooth germ. **(B–E)** Tooth germs at the cap stage of development. Note that p75^NTR^ staining in mesenchyme is localized in the dental mesenchyme (dm) at the early cap stage **(B,C)**, while the staining is mostly seen in the dental follicle (df) at the late cap stage **(D,E)**. Cells from the inner dental epithelium (ide) start to express p75^NTR^, while no staining is detected in the dental papilla (dp) at this late stage **(E)**. Note that nerve fibers (nf) surrounding the dental follicle are strongly stained. **(F)** Higher magnification of the flank area of a tooth germ at the early bell stage of development (Figure 1H). Restricted p75^NTR^ reactivity in undifferentiated cells of the inner dental epithelium. Note that the staining is absent in dental pulp (p). Dotted lines represent the border between the dental epithelial and mesenchymal components. Additional abbreviations: de, dental epithelium; m, mesenchyme; ode, outer dental epithelium; oe, oral epithelium; pa, preameloblasts; po, preodontoblasts; sr, stellate reticulum. Scale bars: 100 μm.

Tooth development proceeds in three well-characterized morphological stages: the bud, cap, and bell stages. At the 5–7th gestational week (g.w.), the epithelial dental buds represent the first epithelial ingrowth into the neural crest-derived mesenchyme at sites corresponding to the position of the future fetal teeth. At that stage, weak NGF immunostaining was evident only in the dental mesenchyme (Figures 1A, 2A), a pattern similar to that observed for TrkA (Figure 1C). While p75^NTR^ labeling was absent from both epithelial and mesenchymal components at the 5th g.w., intense immunoreactivity was seen only in nerve fibers next to the developing tooth germ (Figures 1B, 3A).

**Figure 4:**
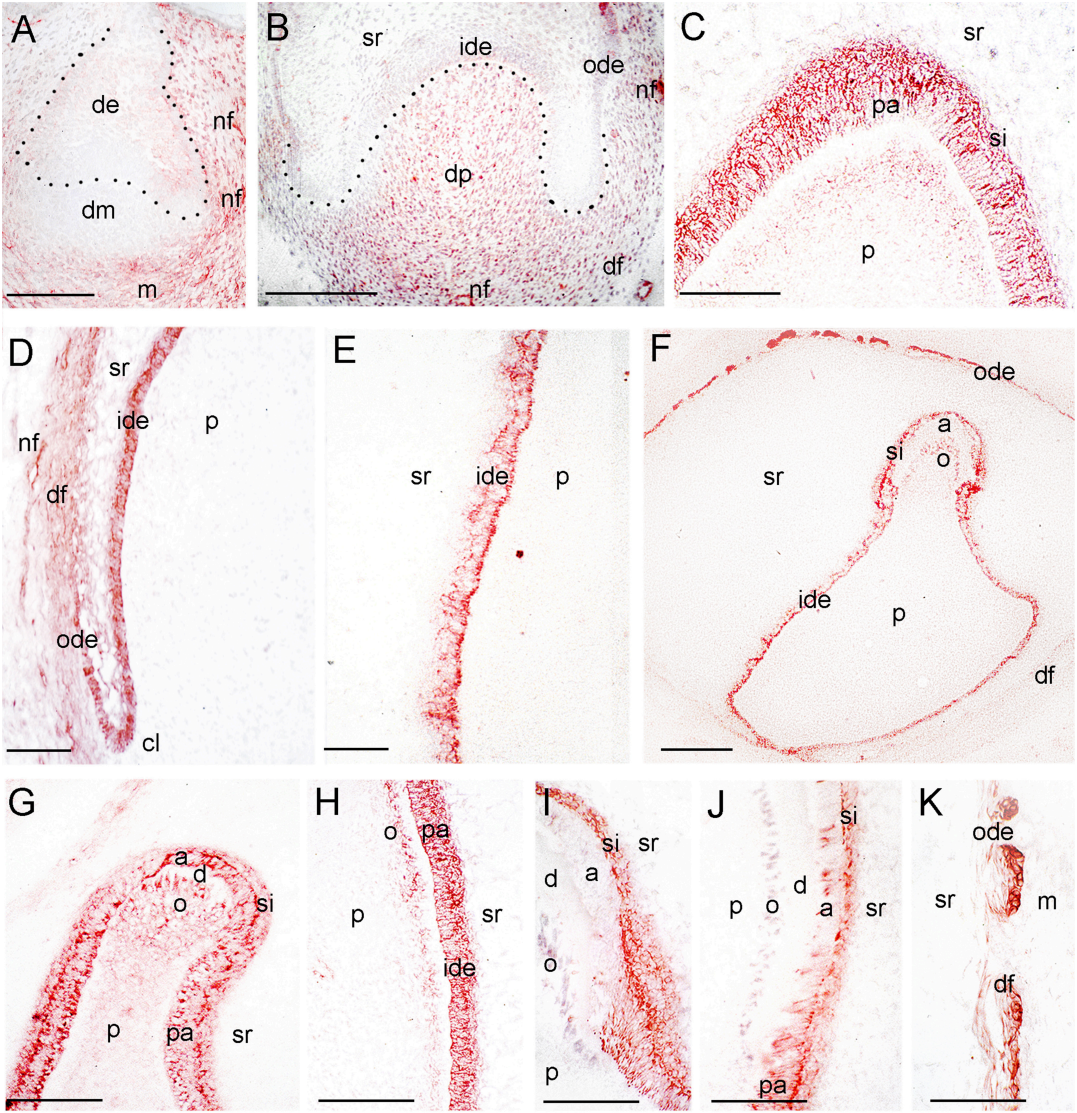
**Expression of TrkA during tooth development. (A)** A tooth germ at the early cap stage of development. TrkA immunoreactivity in red color. **(B)** Higher magnification of a tooth germ at the late cap stage of development (Figure 1F). **(C–E)** Higher magnifications of a tooth germ at the early bell stage of development (Figure 1I), representing the tip of the cusp area **(C)**, the cervical loop and dental follicle territories **(D)**, and an area of the flanks of the forming tooth crown **(E)**. **(F–K)** TrkA staining in tooth germs at the late bell stage of development. **(G–K)** Higher magnifications representing the tip of the cusp areas **(G,I)**, areas of the flanks of the forming tooth crown **(H,J)**, and the dental follicle territory **(K)**. Dotted lines represent the border between the dental epithelial and mesenchymal components. Abbreviations: a, ameloblasts; cl, cervical loop; d, dentinee; de, dental epithelium; df, dental follicle; dm, dental mesenchyme; dp, dental papilla; ide, inner dental epithelium; m, mesenchyme; nf, nerve fibers; o, odontoblasts; ode, outer dental epithelium; p, dental pulp; pa, preameloblasts; si, stratum intermedium; sr, stellate reticulum. Scale bars: **(A,B,F)** 100 μm, **(C–E, G–J)** 25 μm.

Three epithelial cell populations compose the dental epithelium at the cap stage of development (8–15th g.w.): the outer dental epithelium, the inner dental epithelium and the stellate reticulum. The mesenchyme surrounded by the inner dental epithelium gives rise to the dental papilla (future dental pulp), whereas the mesenchyme limiting the dental papilla and encapsulating the enamel organ forms the dental follicle, which gives rise to the supporting tissues of the tooth (future periodontium). During the cap stage, NGF expression was highly dynamic. At early cap stage NGF immunoreactivity was absent from the dental mesenchyme and the adjacent dental epithelium, while it was present in the dental epithelium located far from the mesenchyme (Figure 2B). At the late cap stage, however, NGF was clearly expressed in the inner and outer dental epithelia, in the dental papilla and dental follicle, while expression was very weak in the stellate reticulum (Figure 1D). p75^NTR^ expression was also extremely dynamic at this stage. At the 8th g.w. (early cap stage), intense p75^NTR^ staining was seen in the condensed dental mesenchyme as well as growing nerve axons, while p75^NTR^ staining was absent from all cells of the dental epithelium (Figures 3B,C). At the late cap stage (12th g.w.), p75^NTR^ expression decreased in the dental papilla, while the staining was concentrated in the dental follicle surrounding the dental epithelium (Figure 3D). At 15th g.w., p75^NTR^ labeling was restricted in proliferating cells of the inner dental epithelium, in cell of the dental follicle, as well as in nerve fibers (Figures 1E, 3E). At the early cap stage (8th g.w.), TrkA immunoreactivity was absent from both the dental epithelium and dental mesenchyme, while staining was observed in the mesenchyme surrounding the developing tooth germ (Figure 4A). At the late cap stage (15th g.w.), TrkA staining was localized in the dental papilla, as well as in nerve fibers surrounding the tooth germ (Figures 2F, 4B).

Continuous growth of the tooth germ leads to the bell stage of development (18th–23rd g.w.). The cells of the inner dental epithelium and outer dental epithelium rapidly proliferate in an apical direction and the tooth shape (i.e., crown morphology) is now observable. Dentinogenesis is initiated at the tips of the cusps at the late bell stage (23rd g.w.). Changes occur in peripheral undifferentiated dental pulp cells, which are separated from the inner dental epithelium by a cell-free zone. Mesenchymal cells adjoining this zone polarize, differentiate into odontoblasts and start to secrete the organic matrix of predentinee/dentinee. Changes also occur in the adjacent inner dental epithelial cells that change their shape, stop dividing, polarize, and differentiate into preameloblasts/ameloblasts that assume their enamel-forming function. During the early bell stage (18th g.w.), NGF immunoreactivity was observed in all dental epithelial cells, whereas the labeling was absent from the dental pulp (Figure 1G). p75^NTR^ staining was evident in proliferating cells of the inner dental epithelium but the staining was considerably decreased in preameloblasts at the tip of the cusp (Figures 1H, 3F). Strong TrkA labeling was detected in cells of the inner dental epithelium, preameloblasts, stratum intermedium, and cells of the outer dental epithelium (Figures 1I, 4C–E), while a very weak staining was detected in pulp fibroblasts at the cusp area (Figure 4C). No TrkA immunoreactivity was detected in the stellate reticulum.

At the late bell stage (23rd g.w.) strong NGF staining was observed in undifferentiated (i.e., inner dental epithelium, outer dental epithelium, stellate reticulum, stratum intermedium) and differentiated dental epithelial (i.e., preameloblasts, ameloblasts) as well as in preodontoblasts, and odontoblasts (Figures 2C–E). Undifferentiated cells exhibited a strong NGF labeling on their surface (Figures 2E–G). Weak NGF immunoreactivity was also detected in the dental follicle (Figures 2E–G), while the staining was stronger in nerve fibers surrounding the tooth germ (Figures 2F,G). At this stage, p75^NTR^ immunoreactivity was absent in ameloblasts, odontoblasts, and fibroblasts of the dental pulp, but persisted in undifferentiated inner dental epithelial cells near the cervical loop region (Figure 3F and data not shown). In contrast, TrkA labeling was detected in preameloblasts and differentiating ameloblasts, while the staining was considerably decreased in mature ameloblasts (Figures 4F–J). In the dental pulp, strong TrkA immunoreactivity was observed in differentiating odontoblasts, but this reactivity was significantly reduced in secreting odontoblasts (Figures 4F–J). TrkA immunostaining was also expressed in cells of the dental follicle (Figure 4K).

## Discussion

Sequential and reciprocal interactions between oral epithelium and cranial neural crest-derived mesenchyme result in tooth formation and generation of specific hard tissues, the enamel and dentine (Mitsiadis and Graf, 2009; Jussila and Thesleff, 2012). The present study describes the distribution of NGF, p75^NTR^, and TrkA proteins in the developing human fetal teeth, and confirms that their temporospatial distribution in human dental tissues is very similar with that observed previously in rodents (Yan and Johnson, 1988; Byers et al., 1990; Mitsiadis et al., 1992, 1993; Mitsiadis and Luukko, 1995). NGF is expressed in the epithelium during the early stages of tooth development, while p75^NTR^ and TrkA are first expressed in the mesenchyme. The expression of NGF, TrkA, and p75^NTR^ in the tooth germ significantly precedes the onset of tooth innervation. This pattern of expression suggests a paracrine mode of action of NGF during this stage of odontogenesis and also indicates a role of this molecule in epithelial-mesenchymal interactions. Our present data in humans and previous findings in rodents show p75^NTR^ expression in the condensed mesenchyme of the cap staged molars (Mitsiadis et al., 1992; Mitsiadis and Luukko, 1995). Adhesive functions have been proposed for p75^NTR^ during neuronal development (Chao, 1992; Mirnics et al., 2005), suggesting that this low affinity NGF receptor may be implicated in the condensation of the neural crest-derived mesenchyme and its specification in dental mesenchyme. p75^NTR^ expression in dental follicle coincides with the outgrowths of the maxillary and mandibular nerves around the tooth germs. One of the proposed functions of p75^NTR^ is to increase the local concentrations of NGF (Chao, 1992), thus providing a tropic and trophic support for specific neurons that follow the concentration gradients of NGF.

At later stages of tooth formation, when cytodifferentiation starts, NGF and TrkA are distributed in proliferating cells of the inner dental epithelium, as well as in preameloblasts and the enamel secreting ameloblasts. However, p75^NTR^ expression in dental epithelium is more restricted and confined to undifferentiated, still proliferating, inner dental epithelial cells. The same pattern of p75^NTR^ distribution has been previously detected in human fetal teeth (Christensen et al., 1993) and in the developing rat molars (Byers et al., 1990; Mitsiadis et al., 1992, 1993; Mitsiadis and Luukko, 1995). Concomitant NGF, TrkA, and p75^NTR^ expression in proliferating cells of the inner dental epithelium suggests that NGF may act as a mitogenic factor. In fact, this function of NGF has been already evidenced in the developing human skin (Yaar et al., 1991), human airway smooth muscles (Freund-Michel et al., 2006), and the whisker follicles of the mouse (Davies et al., 1987). During cytodifferentiation, NGF and TrkA are localized around the nucleus and in the apical part of differentiating and functional ameloblasts, whereas p75^NTR^ is not expressed in these cells, thus indicating that NGF can accomplish its biological activities (i.e., differentiation, enamel matrix synthesis, and deposition) in these cells through the TrkA receptor.

In the dental pulp NGF and TrkA are co-expressed in polarizing and differentiated odontoblasts, also indicating that NGF, upon binding to the TrkA receptor, could regulate the function and differentiation of mesenchymal cells, as suggested by previous *in vitro* studies (Arany et al., 2009). The expression pattern of NGF in the pulp of developing human teeth is identical to that previously observed in the pulp of developing rodent teeth (Mitsiadis et al., 1992, 1993; Mitsiadis and Luukko, 1995), illustrating a close correlation between the appearance of NGF and the process of odontoblast differentiation. The present results also confirm prior studies on p75^NTR^ expression in embryonic human teeth, where this low-affinity NGF receptor was absent from dental pulp cells at the bell stage (Christensen et al., 1993). However, in contrast to our previous findings in developing rodent teeth showing p75^NTR^ expression in preodontoblasts and cells of the sub-odontoblastic layer (Mitsiadis et al., 1992, 1993), in human teeth these two cell populations lack p75^NTR^, thus indicating that the mode of NGF action could be variable according to the species.

In the developing human tooth germs, NGF, p75^NTR^, and TrkA are also expressed in the dental follicle (i.e., future periodontium), where mark mainly the nerve fibers. It has been shown that NGF and TrkA play a fundamental role in the innervation and nociception of dental tissues, since mutations in the gene coding for TrkA (i.e., *NTRK1*) leads to pain insensitivity and in some cases to tooth agenesis (Bonkowsky et al., 2003; Gao et al., 2013). In rodents, tooth movement up-regulates NGF, p75^NTR^, and TrkA expression in the periodontal ligament, which is associated with increased sensory nerve fibers sprouting and pain perception (O'Hara et al., 2009). NGF and TrkA expression in the dental follicle indicates that these molecules might modulate nerve growth and sprouting in the periodontal ligament of human teeth in both physiological and pathological conditions. Similarly, NGF expression is up-regulated upon injury in the pulp of mouse molars, leading to massive sprouting of TrkA expressing nerve fibers toward the site of injury (Sarram et al., 1997). The above mentioned findings suggest that innovative strategies could be applied in dental clinics for patients pain relief, since TrkA-positive nerve fibers are important mediators of pain perception in dental tissues (Sullins et al., 2000; Gao et al., 2013; Kyrkanides et al., 2015). Inhibition of NGF/TrkA signaling could be an effective approach to eliminate or reduce pain associated with dental pathologies and clinical interventions. In this context, NGF/TrkA/p75^NTR^ gained enormous attention as potential targets for treatments against cancer-associated pain, leading to the development and trial of several classes of small-molecule-inhibitors and antibodies targeting this signaling axis (Demir et al., 2016).

Apart from its implication in pain perception, the neuro-attractive effect of NGF signaling could be an important modulator of tooth development and regeneration. Innervation plays a key role in the development and the regeneration of orofacial organs (Pagella et al., 2014), and consists one of the fundamental component of stem cell niches (Katayama et al., 2006; Méndez-Ferrer et al., 2008; Knox et al., 2013; Pagella et al., 2015). Both the periodontium and the dental pulp host various stem cell populations that guarantee their repair and regeneration. It has been evidenced on mouse models that tooth injury leads to the activation of NGF signaling and the concomitant attraction of nerve fibers (Sarram et al., 1997; Yamashiro et al., 2000; Kaukua et al., 2014), which might provide necessary but so far unidentified cues for proper formation and regeneration of dental tissues.

**Figure 5:**
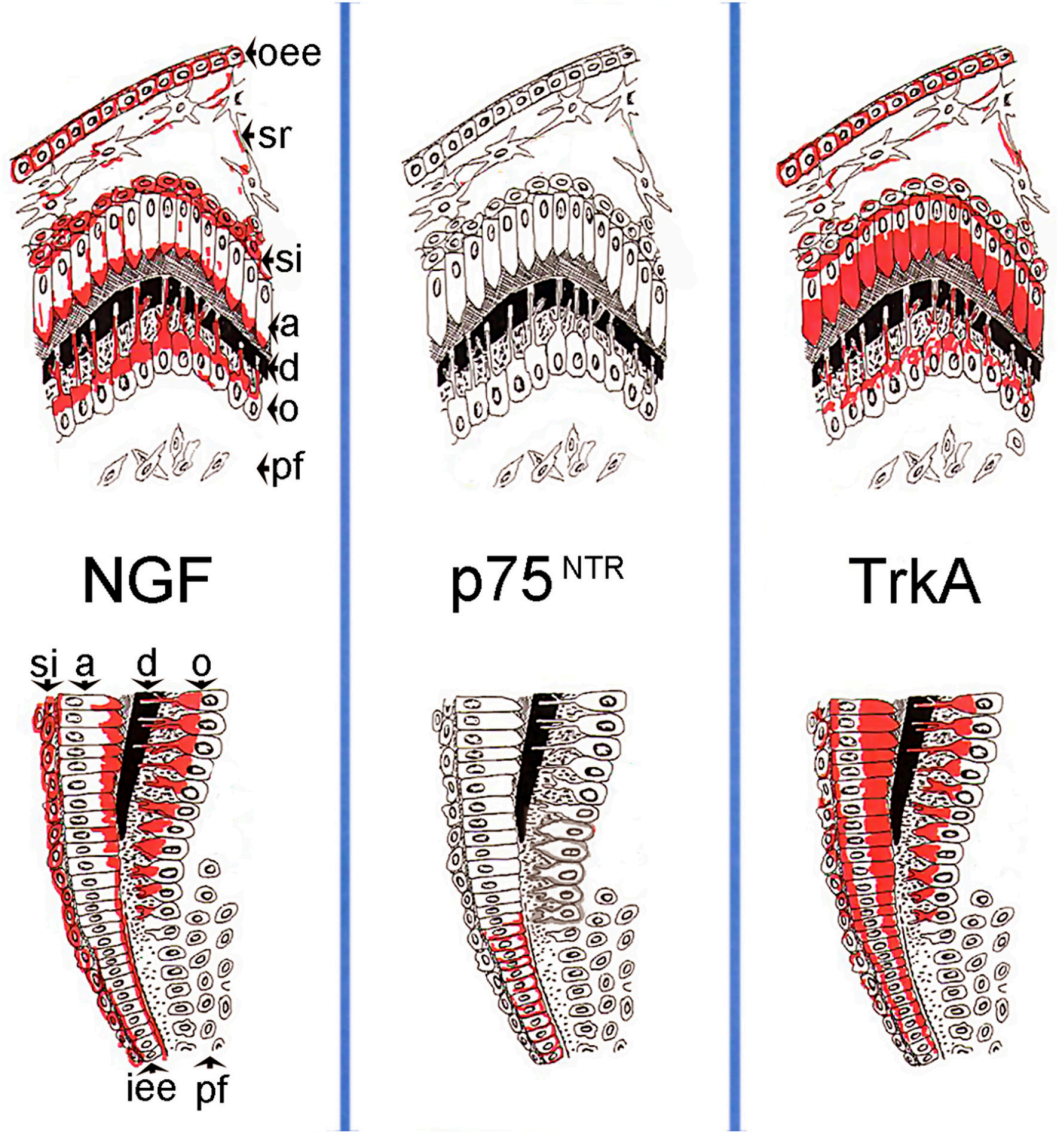
**Schematic illustration of the expression patterns of the NGF, p75^**NTR**^, TrkA proteins in developing human teeth during the differentiation/mineralization events**. On the top is represented the tip of the cusp territory, on the bottom the flank of the tooth crown territory. Abbreviations: oee, outer enamel epithelium; sr, stellate reticulum; si, stratum intermedium; a, ameloblasts; d, dentinee; o, odontoblasts; pf, pulp fibroblasts; iee, inner enamel epithelium.

In conclusion, the present findings suggest a regulatory role for NGF in both mesenchymal and epithelial components of the developing human teeth. NGF, p75^NTR^, and TrkA expression in dental tissues (Figure 5) indicates a function for NGF signaling in cell proliferation, differentiation and mineralization events during odontogenesis, paralleling its role in the development of the nervous system. More precisely, expression of all these three molecules in inner dental epithelial cells correlates with their proliferation status, while co-expression of NGF and TrkA alone in dental epithelial and mesenchymal cells is associated with their differentiation into ameloblasts and odontoblasts. Moreover, expression of the NGF, p75^NTR^, and TrkA proteins in nerve fibers of the developing human teeth indicates that NGF signaling is also involved in their sprouting and attraction toward dental tissues.

## Author contributions

TM and PP equally contributed to the collection of data, analysis of results, writing, and editing of the manuscript.

### Conflict of interest statement

The authors declare that the research was conducted in the absence of any commercial or financial relationships that could be construed as a potential conflict of interest.
